# Cultural Artifacts Transform Embodied Practice: How a Sommelier Card Shapes the Behavior of Dyads Engaged in Wine Tasting

**DOI:** 10.3389/fpsyg.2019.02671

**Published:** 2019-12-06

**Authors:** Joanna Rączaszek-Leonardi, Julia Krzesicka, Natalia Klamann, Karolina Ziembowicz, Michał Denkiewicz, Małgorzata Kukiełka, Julian Zubek

**Affiliations:** ^1^Human Interactivity and Language Lab, Faculty of Psychology, University of Warsaw, Warsaw, Poland; ^2^Faculty of “Artes Liberales”, University of Warsaw, Warsaw, Poland; ^3^College of Inter-Area Individual Studies in the Humanities and Social Sciences, University of Warsaw, Warsaw, Poland; ^4^Institute of Psychology, The Maria Grzegorzewska University, Warsaw, Poland; ^5^Centre of New Technologies, University of Warsaw, Warsaw, Poland

**Keywords:** embodiment, embodied learning, cultural transmission, artifacts, interpersonal coordination

## Abstract

The radical embodied approach to cognition directs researchers’ attention to skilled practice in a structured environment. This means that the structures present in the environment, including structured interactions with others and with artifacts, are put at least on a par with individual cognitive processes in explaining behavior. Both ritualized interactive formats and artifacts can be seen as forms of “external memory,” usually shaped for a particular domain, that constrain skilled practice, perception, and cognition in online behavior and in learning and development. In this paper, we explore how a task involving the recognition of difficult sensory stimuli (wine) by collective systems (dyads) is modified by a domain-specific linguistic artifact (a sommelier card). We point to how using the card changes the way participants explore the stimuli individually, making it more consistent with culturally accrued sommelier know-how, as well as how it transforms the interaction between the participants, creating specific divisions of labor and novel relations. In our exploratory approach, we aim to integrate qualitative methods from anthropology and sociology with quantitative methods from psychology and the dynamical systems approach using both coded behavioral data and automatic movement analysis.

“We join ourselves to the living world by the artifacts of art and science – by made things”
Wendell Berry, *Life is a Miracle*, p. 83

## Introduction

In the ecological psychology approach, the forces shaping behaviors and skills are thought to belong to the structured environments and to individual adaptations and tunings to these environments, with both having equal importance. Behavior is guided by affordances, which are relational properties. Thus, they are neither properties of the world nor of the organism but, rather, are “relations between the abilities of animals and features of the environment” (Chemero, 2003, p. 189). Often in psychological explanations, however, the explanatory thrust is aimed at the individual cognitive processes and structures, while the recognition of the role of environment is left to anthropological or ethnographic studies. Recent developments in embodied, distributed, and situated cognition have changed this situation, resulting in a substantial body of integratory work (Hutchins, 1995, 2006; Cannon-Bowers and Salas, 2001; Fowler et al., 2008; De Jaegher et al., 2010; Cooke et al., 2013; Hasson and Frith, 2016; Fuchs, 2017; Di Paolo et al., 2018). Following and elaborating these approaches, we present a study in which we aimed to recognize the role of an element of the environment, a culturally constructed professional artifact, in shaping individual and collective behavior.

We advocate a systemic approach, in which the structures of the environment (including artifacts and others’ behavior) are treated as constraints on systems’ dynamics, able to influence these dynamics on multiple levels and leading – in specific cases – to new functional organizations. Within this approach, learning individual skills encompasses processes on many time scales and is contingent on organizing through niche construction. Thus, learning on a slower time scale includes making artifacts, such as protocols, tools, machines, and sports equipment, that ratchet the learning process by acting on individuals, shaping their actions and interactions. Such ritualized interactive formats and artifacts can thus be seen as forms of “external memory,” cues to the “right” ways to do things, usually shaped for a particular domain. They constrain skilled practice, perception, cognition, and interaction, allowing for effective collaborative actions across groups and time spans due to their form (i.e., how they are stabilized and designed) and, importantly, due to agents’ tuning-in to social affordances, which enables their use (e.g., Schmidt, 2007; Marsh et al., 2009). Social roles and norms restrict affordances associated with the physical properties of objects by designating who can undertake a certain action on the object and when or which actions are considered appropriate or inappropriate (Schmidt, 2007). The goal of this work is to show the utility of such systemic thinking about artifacts in skillful performance and propose ways to measure their active involvement in shaping both the results and the actions and interactions themselves.

Our study is a follow-up analysis of an earlier study by Zubek et al. (2016), which aimed to measure the collective gain in the recognition of difficult sensory stimuli. The collective gain was expected to result from interaction with another participant and/or with an artifact designed specifically for the description of the stimuli. In that study, the participants learned to recognize and distinguish several wine types, either individually or in pairs, with or without the aid of a sommelier card. The results showed interesting differences in performance between the pairs using the artifact and those not using it. A quantitative approach using bias-variance analysis showed a marked decrease in the variance of responses given by pairs with the card and a slight, albeit non-significant, benefit in performance. In the present research, we focus on the influence that the presence of the card exerted on the process of performing the task. Therefore, we compare the systemic behavior of pairs using the card with that of pairs with no card, analyzing the dyads’ behavior both on the individual and collective levels.

As mentioned above, in our investigation of the sommelier card’s role in collective tasks, we adopt a systemic perspective, striving to integrate psychological, anthropological, and sociological approaches. Therefore, we perceive wine recognition not only as a cognitive task but, equally importantly, as a culturally embodied practice. Likewise, we view the sommelier card not only in terms of its surface linguistic content, processed by individuals, but also as a cultural artifact that embodies the knowledge and experience of past generations of sommeliers and that is itself a structured physical object able to influence individuals and their interactions. There are understandable difficulties with portraying such influences in their full complexity. A standard experimental methodology requires defining variables and measures before performing any experimentation to enable objective judgment of the experimental outcomes. In the original study by Zubek et al. (2016), the interpretation of the two factors – the presence of an interaction partner and the presence of a sommelier card – became difficult because it was not known how the card altered the way in which people worked together. The interactions of the dyads who used the sommelier card could have had an internally different structure and meaning than the interactions of the dyads without the card, raising a question if a simple linear effect of a “presence of interaction” factor captured this change accurately. In the present paper, we combine qualitative intuitions with quantitative analysis to explore this issue. We adopt a dynamical systems theoretical framework, which allows for a pluralistic account of complex phenomena using multiple levels of description (Abney et al., 2014; Witherington, 2015).

In our case, we observe the relevant phenomena on the level of sequences of qualitatively coded individual behaviors, on the level of the coordination of behaviors, and on the level of automatically extracted movement measures. In addition to observing specific changes (e.g., an increase in the frequency of certain behaviors), we strive to reconstruct general systemic properties from a set of interrelated variables participating in the interaction-dominant systemic dynamics (Van Orden et al., 2003). We thus explore the patterns of individual and interactive behavior in terms of their stability, variability, and complexity using specific methods of time series analysis: information-theoretic measures of transition probabilities (Cover and Thomas, 1991; Papapetrou and Kugiumtzis, 2013) and recurrence quantification analysis (Marwan et al., 2007). Using these methods, we are able to describe systems’ dynamics in both qualitative and quantitative terms, including many different facets of the influence of the cultural artifact. We seek to illustrate the versatility of this approach based on the wine recognition task as the model of a cognitive embodied collective task.

The structure of the paper is as follows: In the section below we establish an integrative theoretical framework for considering the role of artifacts in collective tasks. This framework allows us to formulate our research questions, concrete hypotheses, and exploratory goals. In the section “Our Study” we briefly describe the original study, with its analyses and results, and operationalize our questions and hypotheses within its context. The section “Materials and Methods” is devoted to the procedures and methods used in this study for data coding and analysis. The section “Results” describes the outcomes of the analyses on the individual and collective levels. We conclude with a discussion, conclusions, and further prospects.

### The Role of Artifacts in Distributed Cognitive Systems

The embodied, distributed, and situated approaches to cognition, resonating with earlier thought in anthropology and sociology, allow for a change in the conceptualization of objects present in the environment, especially artifacts. In contrast to regarding them as objects of perception whose properties have to be represented in an individual’s knowledge in order to exert their influence on behavior, they can be treated as carefully, culturally shaped constraints, having a much more immediate impact on action and interaction. This reconceptualization of objects with respect to social relations is apparent in recent anthropological and sociological works, which we briefly refer to below. We argue that connecting these works more closely with the ecological psychology views on perception and action, especially with recent developments in the field regarding social affordances, is especially promising in making the picture more complete, resulting in concrete, sensible measures, and testable predictions.

In anthropology, the “agency” of things seems more readily recognized than in most approaches within cognitive psychology. It is acknowledged that things can influence the construction of a human identity and relationships with others and with the environment. They are considered “unknown actors” or “silent things,” i.e., subjects with a kind of agency similar in some respects to that of the owners of a unique subjectivity (Olsen, 2003). Considering the role of cultural artifacts and their agency is crucial for understanding the social world. Going even further, in sociology, Bruno Latour’s actor-network theory describes the world from the perspective of the relations between artifacts and humans, treated equally as actors in the network (Latour, 2005, pp. 46, 52–53). Things can become social actors as long as they influence social reality. Thus, their agency is premised on their presence changing the behavior of their users and the relations among them.

Obviously, the agency of a thing remains incomparable to the agency of a human or, more generally, a living being, as it is crucially dependent on other elements of a cognitive system and its context (Van Oyen, 2018). However, such approaches take us beyond the traditional psychological theoretical tendency to regard objects as passive sources of “input” shaping behavior only because of how they are represented. This prompts more quantitatively oriented researchers to seek operationalizations for the “agency of things” within composite cognitive systems and on multiple time scales.^1^ This is consistent with recent developments toward embodied and distributed theories of cognition, whose approach to the role of artifacts we consider next.

Most of the above accounts are in agreement that an object may have a form of agency only by virtue of its existence within a social reality as it affects the behavior of and relations among the people interacting with it. While anthropological, archeological, and sociological approaches focus on longer time scales, showing how material things shape the way societies and cultures develop and sustain themselves (Ingold, 2013; Malafouris, 2013), cognitive science tends to focus on the online influence on behavior. Within the perspectives of extended (Clark and Chalmers, 1998) and distributed (Hutchins, 1995) cognition, objects are considered constituent parts of cognitive systems. Congruently with the abovementioned sociological approaches, objects can be taken to constitute agentive elements with the ability to change important properties of the system’s organization and relations with the environment. This is possible because artifacts exert specific constraints, enabling or limiting the actions of other elements within a distributed system.

Consider the example of a tightrope walker carrying a balance pole. Carrying the pole increases rotational inertia and lowers the center of gravity. This is beneficial for the walker because these changes in the physical properties of the system make the balancing act easier. To gain this benefit, the walker needs to interact with the artifact appropriately: in this case, the pole must be held steady in the center. The pole may be further adapted for such use by marking its center in some visible way. Thus, the task of tightrope walking is realized by a joint pole-walker system. A walker learning to use the pole is effectively adapting to specific environmental conditions – a form of niche. The pole itself can be adapted (e.g., by marking the center, adjusting the length), which is constructing a cognitive niche (Laland et al., 2000; Clark, 2006) in a way that reflects past walkers’ experience.

A sound theoretical basis for the study of such distributed systems is provided by Hutchins (2010), who proposes viewing human cognition as an activity that arises from the interaction of a cognizer with the social and material environment. Cognition is very often conducted in the presence of other human beings while using various tools – maps, diagrams, tables, calendars, and models of different kinds. These tools are cognitive artifacts (Hutchins, 1999) in the sense that they were created to aid or improve cognition. However, they not only facilitate cognition but are also able to lastingly transform cognition and enable that transformation to become embedded in the culture. A simple, very old example of a cognitive artifact is the abacus, which aids numerical operations such as addition and multiplication. Even though much faster and more precise tools such as calculators already exist, very often schoolchildren are taught a multiplication table with the use of the abacus. The reason is that it can transform multiplication from a simple recollection task to a spatial reasoning exercise, which can later help children to understand notions of area and dimensionality. Although such artifacts are termed “cognitive,” it is the embodied practice that transforms cognition. Tactile interaction with the abacus facilitates embodied understanding of the concept of a measurement unit as well as how to correctly place it in the coordinate system. Another example, given by Hutchins, is culinary art, which is a skill that involves the use of various interactively shared and developed tools – cookbooks, kitchenware, nutrition tables, food-pairing or presentation techniques, and more. A novice cook acquires culinary skills in interaction with these cognitive artifacts, which shape the way cooking is practiced, and in interaction with more experienced chefs using those tools. On the other hand, beginner chefs are encouraged to experiment on their own and come up with novel recipes and practices that may revolutionize the entire field – again, often ratcheted not only by working out new practices but also new artifacts.

**FIGURE 1:**
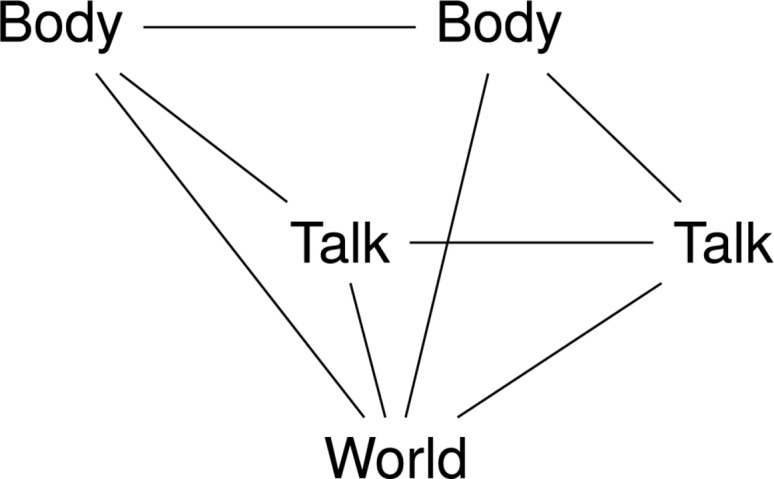
Square-cut gem of interaction (see Hutchins, 2010). In the wine tasting ritual, the sommelier card influences all the relationships specified by the square-cut gem of interaction. It provides language describing the external world of wines’ aroma and flavor that enables tasters to communicate and compare their sensations regarding the wine. Conversely, it also shapes these sensations by providing evaluation categories that the taster would never come up with him- or herself.

The full picture of a “human vs. human vs. object (or artifact)” interaction is captured by the model that Hutchins (2010) calls “a square-cut gem of interaction” (Figure 1). In this view, a multimodal interaction system is distributed across members of a social group whose cognitive process arises from the coordination of their bodies and communication in relation to the external world, including artifacts. Social organization, on a par with the organization of the environment, determines how information is transmitted between group members and thus may itself be viewed as providing architecture for cognition (Hutchins, 2001, 2006). On the other hand, a tool can be incorporated in the way people perceive and control common actions (Hutchins, 2010).

These approaches are very helpful for understanding the roles of niche construction and the distributed and multi-scale nature of cognition involving artifacts, but they usually do not provide details on how artifacts exert their influence. How are behavior and relations affected in each instance of the artifact’s use, and which past, historical processes make such influence possible? Ecological psychology, we believe, is of much help here, as it describes objects, including artifacts, in terms of affordances, relational properties that directly specify the behaviors of cognizing systems. Learning, in this framework, consists in tuning to specific affordances of objects and the niche, changing the behavior of an agent and the agent’s direct perception (e.g., Dreyfus, 2002).

In the case of artifacts, learning involves both an online, individual, as well as collective and social scales when an artifact is embedded into a network of social routines (Schmidt, 2007). Through the predictable re-enactment of the social “game,” objects also gain social meaning, providing for the emergence of particular roles in social interactions. The use of an artifact in a given situation will thus be contingent on culturally evolved social routines. Thus, how an artifact is exerting its power would be excessively difficult to explain when resorting only to the individual’s knowledge regarding how to use it. Much of the artifact’s power lies in – often tacit – reactivation of specific behaviors in social routines, influencing the projects we are constantly engaged in (Merleau-Ponty, 1962). These are real physical events occurring in concrete situations and should be visible as constraints, both on the individual behavioral dynamics and at the level of collective systems. Moreover, the effects of these constraints can be assessed quantitatively with respect to how the presence of an artifact impinges on the behavior of a system: this consists in comparing a distributed system without and with an artifact in terms of the organization of its elements and of the whole. Such understanding creates the possibility of studying the “agency of things” in a more structured and quantitative way, using the methodology of complex systems. Changes can be captured over multiple time scales: slower ones, when social relations are shaped into strands of organized activities and artifacts are shaped through design, and faster, online processes, when the presence of the artifact can directly impinge on perception and action.

In our study, we will use this framework to investigate the online influence of a sommelier card on distributed cognitive systems for recognizing wines. The card is treated as an enabling constraint whose physical presence impinges on individual behavior and shapes interactions through the physical and social meaning it carries, effectuating measurable changes through the presentation of physical and social affordances. We will seek to measure and explore the changes in the distributed cognitive system, studying the organizational properties of its elements and of the whole rather than delving into the cognitive processes and representations of individuals. We believe this to be an informative complementary perspective to gain insights into the cognitive properties of the novel distributed systems created by people interacting with and without an artifact. We also ask if there is a way to gauge the “agency” of the sommelier card both through its effect on the individual participants and through its influence on the relations among the system elements and thus the entire system organization.

### Our Study

In this work, we analyze a subset of results from the previous study of Zubek et al. (2016), which concerned the impact of language on performance in a perceptual learning and recognition task. In that study, language influence consisted of the online linguistic interaction of the participants as well as their interaction with a sommelier card, which is a culturally designed professional linguistic artifact for wine description and recognition. The card was a slightly simplified Polish version of the Associazione Italiana Sommelier card, which, for several years, has been used among the Polish sommeliers. This means that the key dimensions used in the card had the Polish terms agreed upon by the Polish sommeliers and used in professional writing (for details, see Zubek et al., 2016). The task of the participants was first to learn the taste (and smell) of three wine samples and then to recognize these wines among a larger set of wines. The experiment had a classic 2 by 2 factorial design: the participants performed the task either individually or in pairs (free online linguistic interaction) and with or without the aid of a sommelier card (constrained linguistic interaction). Accuracy of recognition in each condition, as well as the structure of errors, was studied as a function of the type of “cognitive system” created in each condition: individual, individual with card, dyad, and dyad with card.

In terms of task performance, Zubek et al. (2016) observed that while recognition as such was mostly unaffected, bias-variance decomposition (Gigerenzer and Brighton, 2009) revealed differences between the groups. In pairs that used the sommelier card, the variance component of the error was decreased compared to that in the other groups. Moreover, the behavior of participants performing the task solo seemed to be largely unaffected by the card. Following the analyses of Fusaroli et al. (2012), the authors of the 2016 study performed linguistic analyses of verbal interactions between the participants. They analyzed the vocabulary used by the participants, focusing on the words used to describe the wine. The analysis revealed that the vocabularies of group members using the card had more words in common than those of participants in the “no card” group, and these vocabularies were more concise, as indicated by the smaller type-to-token ratio (i.e., the ratio of vocabulary size to all uttered words).

Zubek et al. (2016) thus demonstrated that the presence of the card had a structuring influence in streamlining people’s vocabulary and decreasing the variance in dyads only. This prompted us to look more closely at the effects of the sommelier card on the joint behavior of the participants. Consequently, in the present study, we focus on the activity of performing the task itself rather than on participants’ wine-identification performance. We see this activity as a goal-driven, embodied interaction between two people in two conditions: an unstructured interaction and an interaction structured by the sommelier card. Integrating the psychological and anthropological approaches to cultural artifacts, we acknowledge the agentivity of a card as an element of a distributed cognitive system, operationalized as its ability to change individual behaviors and create novel relations among participants. Adopting a dynamic and systemic perspective allows us to measure this constraining influence quantitatively as a change in individual cognitive systems embedded in a larger collective system. The influence can be gauged in terms of ordering based on (i) the timing and coupling of qualitatively coded behaviors at the individual and dyadic level and (ii) more global measures of automatically coded motion patterns.

### Hypotheses

The sommelier card is a cultural artifact, a condensate of knowledge and practical experience exerting constraints on wine tasting practice and the product of the expertise of generations of sommeliers. It contains vocabulary that streamlines the talk of the participants, but it also contains an implicit structure, such as the ordering of sensory descriptions (from visual to olfactory to taste characteristics). It is also a physical object in the cognitive system to which both members of the system can refer. It is thus a constraint that can work on several levels of organization, changing the embodied individual and interactive behavior of the participants and the relation between them in a larger collective system.

Adopting a dynamical systems perspective, it is useful to think about the relevant cognitive systems in interaction with the card in terms of the degrees of freedom and constraints that it may impose. Each system has a characteristic number of degrees of freedom, which means that its behavior may vary freely in certain dimensions. Adding constraints should decrease the number of degrees of freedom, which may introduce order in the behavior, consequently reducing the system’s variability, or which may allow new stable behaviors to appear, thus increasing the system’s variability (so-called enabling constraints). Thus, our general analytical strategy is to measure the behavior of systems with and without a card and to compare their variability and the coupling strength of their elements in various dimensions related to the wine recognition task and communication between participants.

The artifact can influence individuals’ strategies for exploring the wine to be recognized, perhaps prompting them to fall into the patterns of more skilled sommeliers. We thus expect a change in the frequency distribution of exploratory behaviors and their ordering (frequency of transitions). At the collective level, the card, as a physical object in a shared space, may impose additional constraints on the strength of coupling of behaviors. In both cases, we expect more ordering and thus lower entropy of behavior, both as analyzed on the level of meaningful actions and on the level of the interactants’ movements.

More concretely, at the individual level, the proportions of each event (action) type are expected to gravitate toward the patterns exhibited by more experienced sommeliers. The visual modality was not available to the participants as a recognition cue (in the learning phase, wine was served in black wine glasses to minimize reliance on color, as this would render the task too easy); thus, we expect (i) a change in the proportion of drinking vs. smelling behaviors (the latter is required by the wine descriptors included in the card but is not typical for amateur tasters). Moreover, we also expect (ii) a change in the sequences of actions, i.e., which type of event is likely to follow another. While it is difficult to state beforehand what transitions in particular will increase or decrease in frequency, we assume that in the absence of the artifact, the sequences will be more unstructured, resulting in a more uniform distribution of transition types. The card, on the other hand, is expected to induce the participants to repeat certain sequences of events more often than others, the ordering within sequences being influenced by the ordering of the modalities on the card. We are open also to the possibility that the coding process, which forces detailed observation of the qualitative aspects of the participants’ actions, can bring further insights and research questions.

At the collective level, we should also see the structuring effect of the artifact. Here, however, our research is more exploratory. In general, the card, as one more physical element in the shared conceptual and physical space, could act as an additional constraint, introducing order in the behavior of a system as a whole and particular types of coupling (such as the emergence of leader-follower dynamics, where one participant becomes the reader of the card and initiator of behaviors). This may be observed at the level of the analysis of correlative structures of behavior as well as at the level of physical movement, which often reflects such social structures (see Fowler et al., 2008; Paxton and Dale, 2013). Conversely, a card can act as an enabling constraint facilitating the division of labor, such as the “delegation” of a certain modality to a single member of the dyad or the delegation of a card-reader. We explored these possibilities and related the properties of the dyads as systems both to their performance and to the participants’ satisfaction with the interaction.

## Materials and Methods

### Procedure

The experiment was conducted following the ethical guidelines for psychological research and approved by the local ethical committee of the Institute of Psychology, Polish Academy of Sciences. The participants, upon arriving, were assigned to one of the four experimental conditions: individual or dyad, with or without a sommelier card. The assignment was random, the pairs themselves were created by convenience from the participants who were available (in most cases, the participants in the dyads did not know each other; see Table 1). For both the individuals and the dyads, the task was to learn the smell and taste of three different wines (learning phase), and later, after a 40-min break filled with non-verbal tasks, to recognize these wines among other wines (recognition phase). In the learning phase, the three target wines were presented in black glasses labeled 1, 2, and 3; the labeling was consistent for both participants in the pair. The instruction was to remember the wines for the recognition task, which will take place later. Moreover, in this phase (and only in the condition with the cards), three copies of a sommelier card were introduced (see Supplementary Materials). The participants had to fill them out with descriptions of the three wines. The participants were instructed to rely on their own, colloquial understanding of the terms on the card; no additional explanation was provided. In the recognition phase, the participants were given six wine samples (coded A to E; coding was consistent within each pair), among which the initial three target wines were present. They were to point them out and mark them with their original number codes. In the card condition, they were also to match the sommelier cards they had filled out with the respective sample. There was no time limit in either phase. The pairs performed both phases together, jointly filling out one sommelier card per target wine in the learning phase; they were also required to give a single joint answer in the recognition phase. The entire procedure outlined above was explained to the participants at the beginning of the experiment, and the instructions were repeated at the beginning of the recognition phase. Joint sessions were recorded using a video camera and voice recorder. After the experiment, the participants completed a questionnaire containing questions pertaining to their demographic information, perceived quality of cooperation, how well they knew their partner, and other issues (the complete questionnaire translated into English is available in Supplementary Materials). A more in-depth description of the procedure and the reasoning behind it and other details of the experiment can be found in the original article (Zubek et al., 2016, and its Supplementary Materials).

**TABLE 1:** Characteristics of the participants in the dyadic condition in the Zubek et al. (2016) study.

	**Sex**		**Dyad composition**			**Age (years)**			**Acquaintance level**
	**Female**	**Male**	**Both female**	**Both male**	**Mixed**	**Range**	**Avg.**	**Avg. difference**	**No. of pairs of strangers**
Without card	27	15	10	4	7	18–35	22.2	3.0	12
With card	28	10	11	2	6	18–40	23.0	3.4	15
All dyads	55	25	21	6	13	18–40	22.6	3.2	27

*“Avg.” means average. Avg. difference pertains to the average difference in age between the members of the dyad. Acquaintance level was indicated on a scale from 1 (“strangers”) to 4 (“knows partner very well”). The number of dyads in which at least one person indicated they were unknown to their partner is given in the column “No. of pairs of strangers.”*

A total of 123 participants (among them 85 females and one participant who did not state their sex) took part in the experiment. Participants were recruited by advertisements through social media and screened for any conditions that would put them or the quality of the study at risk: contraindications to the consumption of alcohol, smell or taste disorders, professional or advanced knowledge of wines, high frequency of wine consumption, and lack of fluency in the Polish language. Due to the possible effects of advanced age on olfaction (Doty, 1989; Hummel et al., 2007), we also decided to recruit only participants younger than 50 years of age. Altogether, there were 19 pairs with a card^2^, 21 without, and 20 solo participants with and 20 without a card. The demographic characteristics of the participants in both conditions are provided in Table 1.

In this paper, we present an analysis of the second phase of the experiment (recognition phase) focusing on the dyads. It covers material consisting of 40 videos (one for each dyad), comprising a total of 391 min 15 s of video material. The average duration of a recording is 9 min 46 s, and the median is 8 min 56 s. The shortest recording is 3 min 17 s; the longest is 22 min 41 s.

### Data Coding and Analyses

We employed a dual approach to quantify the individual behavior and interaction in each group: manual coding using raters to code the behaviors and automatic coding using software to trace and quantify the movements from the video recordings. We used the ELAN, 2018 (versions 4.9.4 and 5.4, 2016–2018; see also Wittenburg et al., 2006) program to code the timing and type of each relevant behavior. We observed seven main behaviors that constituted vital elements of performing the task: “drinking wine,” “smelling wine,” “drinking water,” “holding cup of water,” “holding cup of wine,” “marking cup,” and “changing cup.” Obviously, this selection of coded behaviors does not include all the behavioral categories that could be coded in such a task situation, such as, for example, gaze direction, speech or the participants’ interaction with the card. We decided to further focus only on four categories from the ones listed above that were directly associated with performing the task and that recurred frequently enough to allow for discerning patterns: “drinking wine,” “drinking water,” “smelling wine,” and “changing cup.”

**FIGURE 2:**
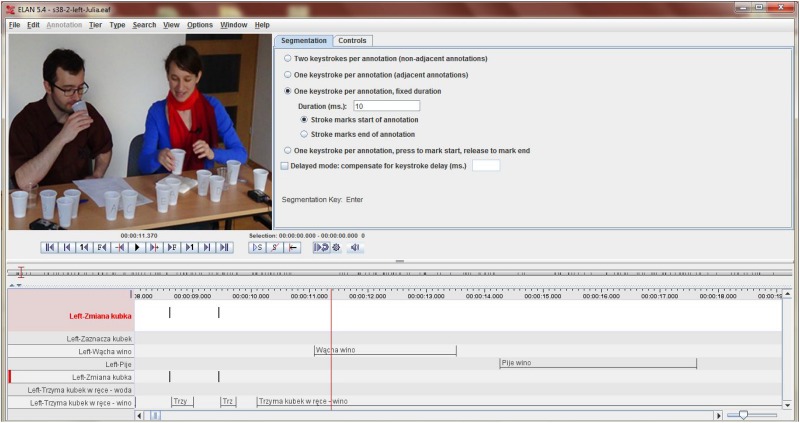
ELAN screen for coding the timing and categories of behaviors. Written informed consent was obtained from the individuals for the publication of this image.

“Drinking wine” was defined as an action of drinking wine from a single cup in a single or prolonged manner, and, similarly, “smelling wine” was smelling – in a short or prolonged manner – from a single cup. In addition to drinking and smelling wine, we also coded such movements as drinking water that is an important part of the professional wine-tasting process. In the case of smelling or drinking, the action began when the cup was held next to the nose or mouth and ended at the first moment in which it was taken away. “Changing cup” was coded when the change of focus occurred “physically” (taking a new cup in hand) or “mentally” (e.g., holding two cups at the same time and changing focus from one to the other or pointing to another cup that was not being held at that moment). All categories were coded as time segments (with a beginning and an end), and only “changing cup” was a point event (as changing focus from one wine to another could occur in several, uncomparable ways, its duration was not taken into account). Figure 2 shows a screenshot from the coding of the videos in ELAN.

One should note that coding behavioral data involves multiple simplifications. While coding, we discovered the richness of real actions and interactions that could not be captured by our simplified coding schema. We discovered unusual behaviors, such as the action of bending over a cup of wine instead of bringing it to the nose, or actions directed toward the other participant, such as smelling wine from another participant’s cup. Another complexity is the ambidextrousness of some actions. A good example of this is the action of smelling two wines at once, using both hands. Even more difficult was defining the level of intentionality in the movements. “Changing cup” was one of the most problematic categories, as the coders had to interpret the participants’ behavior and evaluate whether the movement was indeed made intentionally and purposefully. Finally, some of the participants tended to perform semi-professional movements, such as swirling the wine to raise the fragrance. These behaviors require deeper investigation and may be analyzed in further research. Ultimately, repetitive interaction with the raw empirical data helped us to improve our coding schema^3^ and broaden our understanding of the coding categories and their relation to the videotaped behaviors. A meticulous coding process forces detailed observation, which brings into focus the qualitative aspects of the participants’ actions and can be a source of further hypotheses and investigations. In this respect, we were guided not only by the hypotheses we advanced but by a more qualitative exploratory approach.

Behaviors were coded by four coders. A total of 2.5% of the videos were coded by all of the coders to check for coding reliability. The reliability of the coding was assessed using the *Staccato* algorithm – *Segmentation Agreement Calculator according to Thomann* – a tool for evaluating the reliability of video data annotations, designed specifically for evaluating the reliability of gesture annotations (Lücking et al., 2011). The reliability was calculated based on a 10-min 51-s video sample coded by all four coders. We set default algorithm parameters, which include the number of Monte Carlo iterations (1000), nomination length granularity (10), and the level of significance to reject the null hypothesis of chance-based agreement (0.05). The results are given in the form of *the degree of organisation* parameter (which takes values in the interval [−1, 1]). The overall average degree of organization for all coding categories was 0.78. For “wine drinking,” the overall average degree of organization was 0.96, and for “smells wine,” 0.96, which is close to complete consistency (i.e., to maximum value of degree of organization; see Thomann, 2001; Lücking et al., 2011, for detailed description of the method).

The automatic movement extraction and coding consisted of movement quantification analysis performed using the frame-differencing method (Paxton and Dale, 2013). The frame-differencing method codes movement as a change of pixel color. By comparing the values of pixels in two subsequent video frames, the overall movement of an object in that moment can be measured. The method requires the background of the analyzed object to be static and the regions of interest occupied by each participant to be specified. Using our developed PixelTracking software^4^, we manually specified two non-overlapping regions of interest and extracted time series describing changes in the movement of each participant. The data were then normalized, and a second-order Butterworth low-pass filter was applied to prevent the false detection of participants’ movements caused by fluctuations in light sources (Paxton and Dale, 2013).

These time series were analyzed further using cross-recurrence quantification analysis. Cross-recurrence quantification analysis is a non-linear technique that uses reconstruction of a phase space to analyze the trajectories of two systems (Zbilut et al., 1998). It quantifies the number and duration of occurrences of revisitation of the same state in the state space (given a specified similarity radius) by the analyzed systems, thus providing better insight into their temporal organization and codependency. Applying this method to the data extracted from the video recordings required choosing the following CRQA parameters: radius, delay, and embedding dimension. To this end, we used heuristics implemented in the R package “crqa” (Coco and Dale, 2014) and applied the optimizeParam function to small slices (750 frames) of time series from each session. As a result, the following parameters were chosen: radius = 0.20 (value averaged over all sessions, standard deviation equal to 0.11), delay = 18 (maximum value over all sessions chosen to prevent information loss), and embedding dimension = 2 (the same value was obtained for every session). These parameters were then used to analyze the time series in Commandline Recurrence Plots (Norbert Marwan, ver 1.13, 2006). For each session, the program calculated a cross-recurrence plot that was used to obtain the following measures: determinism, recurrence rate, determinism-recurrence rate ratio, laminarity and the longest vertical line. The statistics were averaged over windows (size – 750, step – 35) along the main diagonal. Additionally, we calculated the absolute amount of movement in a dyad and the difference between the amount of movement of the persons forming a dyad. Thus, each session was characterized by seven movement statistics (see Coco and Dale, 2014, for a detailed description):

• recurrence rate (RR) – how often participants visited similar states, i.e., coordinated movement (probability of recurrence),
• determinism (DET) – how often coordination occurred in prolonged episodes (conditional probability of a prolonged recurrence),
• the ratio between them (DET/RR) – conditional probability of a prolonged recurrence relative to the overall probability of recurrence,
• laminarity (LAM) – how often one system stays for some time in a state visited by the other system (in our case staying in the same state equals to producing movement with constant characteristics),
• longest vertical line (V_max) – the longest episode during which one system has stayed in a state that has been visited by another system,
• the absolute amount of movement in a dyad (abs), and
• the difference between the amount of movement of individuals in a dyad (abs_diff).

Finally, in addition to the complex measures presented above, we made use of the measures from the original study: performance (number of wines correctly identified) and the subjective assessment of the quality of cooperation, self-reported by each participant after the experiment (“How do you assess the quality of the cooperation during the task?”) on a scale from 1 (“low”) to 7 (“very high”) and averaged within each pair.

All raw data used in the analyses (coded behaviors, raw movement signals, and experiment results) are available in Supplementary Materials.

## Results

### Behavioral Event Frequencies

**FIGURE 3:**
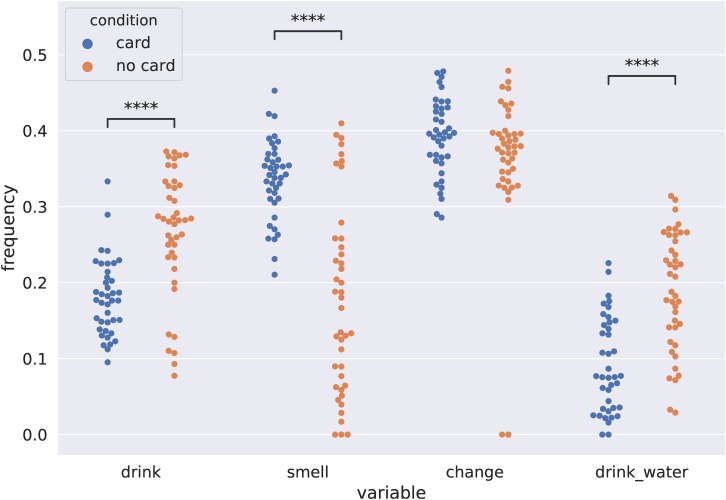
Distributions of event frequencies within the experimental sessions for the two conditions. Each point corresponds to a frequency of a particular event in a single session. Asterisks correspond to significant differences according to Welch’s *t* test with the false discovery rate controlled (^****^p < 0.001).

Using our behavioral coding, we compared the events’ frequencies between the two conditions at the level of individual participants. Figure 3 presents behavior frequencies for the following coded behaviors: “drink” (drinking wine from a single cup), “smell” (smelling a single cup), “change” (picking up a different cup), and “drink_water” (drinking water). To calculate the statistical significance of group differences, we performed four Welch’s *t* tests. *p* values were adjusted using the Benjamini–Hochberg procedure (Benjamini and Hochberg, 1995) to control the false discovery rate in the case of multiple testing. Drinking wine occurred more frequently in the “no card” condition (*t* = −5.61, df = 62.72, *p* < 0.001, *p*_adj_ < 0.001), drinking water was more frequent in the “no card” condition (*t* = −6.06, df = 76.45, *p* < 0.001, *p*_adj_ < 0.001), and smelling wine was more frequent in the “card” condition (*t* = 5.94, df = 62.79, *p* < 0.001, *p*_adj_ < 0.001).

#### Behavioral Event Transition Probabilities

The structure of event sequences was compared in the two conditions. For each session, transitional probabilities were computed, which determined how many events of each type occurred, given the type of preceding event (the event sequences of both participants were used to calculate a single set of transitional probabilities). Repeated occurrences of the same event were excluded (for example, the event sequence “drink”–“drink” was treated as a single occurrence of “drink”). To prevent the counts of events (which – as seen from the previous analysis – were different between the conditions) from impinging on the assessment of differences in transitional probabilities, we calculated mutual information (MI) between consecutive events, which is normalized with respect to the probabilities of single events (Cover and Thomas, 1991; Papapetrou and Kugiumtzis, 2013). MI scores were smaller in the “card” condition than in the “no card” condition (Student’s *t* test, *t* = −2.46, df = 39, *p* = 0.017). This means that, contrary to our hypothesis, the sequence is less structured overall in the “card” condition: knowing a previous event provides less information on the next event.

**FIGURE 4:**
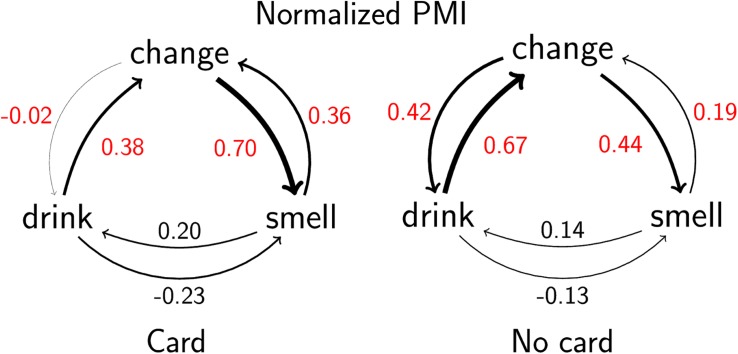
Mean values of normalized pointwise mutual information (PMI) scores for event transitions in the two conditions. Values significantly different between conditions are printed in red.

To better understand this result and to detect possible sequence differences between the conditions, we analyzed the transition probabilities for individual event pairs. We calculated the normalized pointwise mutual information score (normalized PMI, Figure 4), which is positive when two events co-occur together more often than expected considering their base frequencies, negative when two events co-occur less frequently than expected, and zero if events are independent. Normalized PMI is restricted to the interval [−1,1]. There was one session in which the change–smell transition did not occur, and in three sessions, the smell–change transition was not present, which resulted in missing values.

Calculated normalized PMI scores were then compared between conditions (with or without a card) at the level of a session using a series of Student’s *t* tests with Benjamini–Hochberg corrections. The results showed several differences between the behavior sequences in dyads working with and without the sommelier card. We established that drinking immediately after the cup change was less prominent among pairs with the card (*t* = −5.66, df = 38, *p* < 0.001, *p*_adj_ < 0.001), while smelling after a cup change was more prominent (*t* = 4.85, df = 37, *p* < 0.001, *p*_adj_ < 0.001). There were also differences regarding the events preceding a cup change: drinking was less prominent (*t* = −5.40, df = 38, *p* < 0.001, *p*_adj_ < 0.001) and smelling was more prominent (*t* = 2.27, df = 35, *p* = 0.030, *p*_adj_ = 0.045) in pairs working with the card. Consequently, while pairs without the card had a similar tendency to start an interaction with a new wine by drinking or smelling it, pairs with the card favored smelling. Similarly, pairs without the card ended their interaction with a wine more often by drinking, but among pairs with the card, drinking and smelling were equally prominent. Generally, we may conclude that introducing the sommelier card opened up new possibilities for interacting with the wine via the olfactory modality, even when the base frequencies of events were accounted for.

### Behavioral Coordination Within Pairs

We demonstrated that the presence of the sommelier card significantly altered the behavior of individuals. According to our hypotheses, it should also influence the coordination between participants working together. As a first step, we compared behavior frequencies within pairs. For each pair, we calculated the absolute difference between the observed frequencies of a particular behavior between the two participants. The differences were small (mean difference 0.029, max 0.136), and no significant differences between the “card” and “no card” groups, according to Welch’s *t* test, were observed (the statistics for specific events were as follows: drink – *t* = −0.51, df = 38.74, *p* = 0.612, smell – *t* = −0.64, df = 38.95, *p* = 0.523, change – *t* = −0.23, df = 38.67, *p* = 0.821, drink_water – *t* = -0.07, df = 37.54, *p* = 0.945).

**FIGURE 5:**
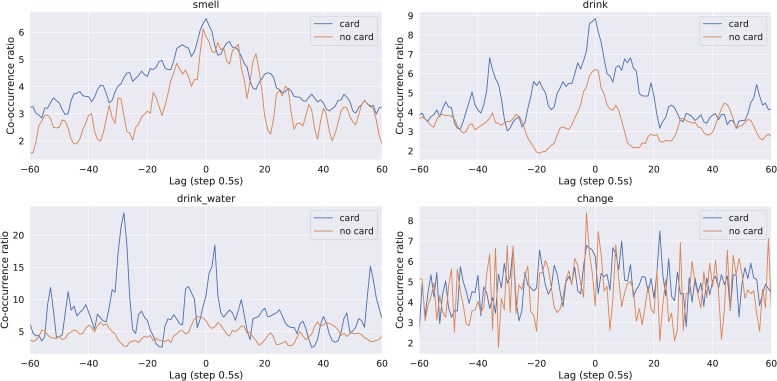
Observed coordination structures for different events in the two groups for the four coded behaviors. Averaged time-lagged profiles of the co-occurrence ratio are presented for each event separately.

**FIGURE 6:**
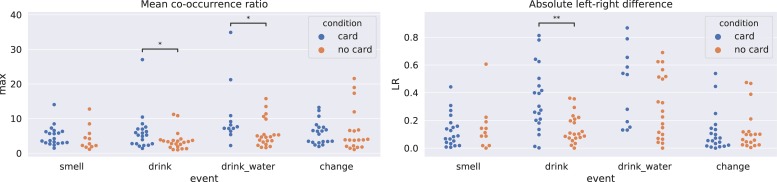
Distributions of the maximal co-occurrence ratio observed (for any lag) and the absolute difference between the left and right side of the profile. Values are calculated separately for each event category. Significant differences between the two groups according to the Mann–Whitney *U* test are marked with asterisks (^∗^*p* < 0.05, ^∗∗^*p* < 0.01).

To gauge patterns of behavioral coordination between the two participants within pairs, we discretized time in our sequence of behaviors and obtained standardized time series with a sampling frequency of 0.5 s. For a specified time lag *l*, we calculated probability *p*(A*_*t*_*, B*_*t*_*_+l_) that if participant A performs an action at time *t*, participant B performs the same action at time *t* + *l*. Positive lag corresponds to participant A leading and B following, and negative lag to the opposite scenario. The obtained probabilities were normalized by dividing them by the baseline value (the probability of the two events occurring in this configuration at random) to obtain co-occurrence ratio *r* = *p*(A*_*t*_*, B*_*t*_*_+l_)/*p*(A*_*t*_*)/*p*(B*_*t*_*_+l_). The co-occurrence ratio was defined only when the considered event occurred at least 10 times for each participant. We focused our analysis on lags ranging from −25 steps to 25 steps (−12.5 s to 12.5 s), because maximal values of the co-occurrence ratio were observed in this time window. The characteristics of each profile were further aggregated into two values: the mean observed co-occurrence ratio within the profile for lags [-25,25] (Figure 5) and the normalized difference between the left and right sides of the central profile (*d* = |*L*−*R*| /(*L* + *R*), where *L* is the sum of ratios for lags [−25,0) and *R* is the sum of ratios for lags (0, 25]). The mean co-occurrence ratio might be interpreted as the mean amount of structured coordination observed for the pair. Left–right (LR) difference describes the amount of asymmetry in the roles in the interaction: a larger LR difference means that one participant consistently leads and the other follows. These two measures calculated for each pair separately were used in further analyses. Figure 6 presents the coordination statistics for different behaviors.

Because of the irregular shape of the distributions, the significance of these results was calculated using the Mann–Whitney *U* test to compare the distributions of the maximum ratio and LR difference between the two groups. The false discovery rate was controlled using the Benjamini–Hochberg correction. When drinking wine, pairs with a card displayed a greater amount of role asymmetry, as shown by the LR difference (*U* = 330.0, *p* = 0.002, *p*_adj_ = 0.016), and a trend toward stronger coordination below the level of significance (*U* = 290.0, *p* = 0.038, *p*_adj_ = 0.101). For drinking water, we observed a trend toward greater coordination in the group with the card (*U* = 190.0, *p* = 0.017, *p*_adj_ = 0.068), although the effect did not reach statistical significance after adjusting for multiple comparisons. These results indicate that in the “card” condition, participants were coordinated in a particular way when drinking, and stable leader–follower roles emerged. Neither of these characteristics of the pairs correlated with task performance or reported satisfaction with the collaboration (*F* test for the linear model predicting task performance based on behavioral coordination statistics: *F* = 0.81, df = 4;34, *p* = 0.525, *F* test for the linear model predicting satisfaction based on behavioral coordination statistics: *F* = 0.52, df = 4;34, *p* = 0.718).

### Low-Level Movement Coordination

We were also interested in how card-dependent patterns of behavior, which were quantified via the behavioral coding, translate into differences in the low-level movement properties of the interacting dyads, obtained from the frame-difference method and cRQA analysis.

To confirm that the obtained cRQA statistics are non-trivial and capture real variability in behavior, we analyzed the data using a pseudosynchrony paradigm (Bernieri et al., 1988). Time series describing the total movement of one subject from each session were paired with the movements of a person from a different session, thus allowing us to compare the results with a baseline obtained from randomly assigned pairs. This baseline allowed us to distinguish between false coordination arising from the task structure (for example, the natural sequence of wine tasting followed in every session) and true interpersonal coordination. We compared the values of the cRQA measures between 40 real pairs and 40 generated artificial pairs using the Welch *t* test (the false discovery rate was controlled using the Benjamini–Hochberg correction). The results are given in Table 2. For three statistics (RR, DET, and DET/RR), we found significant differences; the other two (V_max and LAM) did not reach significance. Overall, cRQA statistics are able to capture the difference between the coordination of real pairs and that of artificial pairs.

**TABLE 2:** Welch *t* test results for the comparison of cRQA statistics between real and artificial pairs (pseudosynchrony).

**cRQA measure**	***t***	**df**	***p***	***p*_adj_**
Recurrence rate (RR)	3.47	46.47	0.001	0.005
Determinism (DET)	2.99	73.24	0.004	0.010
Determinism to recurrence rate ratio (DET/RR)	−2.39	77.18	0.019	0.031
Longest vertical line (V_max)	1.42	72.20	0.160	0.160
Laminarity (LAM)	1.58	77.20	0.118	0.148

As a next step, we compared the overall movement coordination statistics between the “card” and “no card” conditions. We used logistic regression to determine whether the movement characteristics allow for prediction of the condition in which the task was performed (with or without a card).^5^ All of the cRQA measures mentioned before were used as predictors: determinism (DET), recurrence rate (RR), the ratio between them (DET/RR), laminarity (LAM), and longest vertical line (V_max), as well as the absolute amount of movement in a dyad (abs), the difference between the amount of movement of individuals in a dyad (abs_diff) and reported satisfaction with cooperation. The model was compared with a null model using the likelihood ratio test, and the outcome was not significant (model log-likelihood LL = −27.68, χ^2^ = 11.859, df = 8, *p* = 0.158), thus not supporting such a prediction.

We also determined whether the relations between movement coordination and two other variables, task performance and reported satisfaction with collaboration, depend on the presence of the sommelier card. For each dependent variable, two separate linear regression models were created: one for the group with the card and one for the group without the card. We tested overall model significance using *F* tests. The relationship between movement characteristics and reported satisfaction was significant in the group working with the card (*F* = 5.631, df = 7;11, *p* = 0.006) and not significant in the group without the card (*F* = 0.953, df = 7;13, *p* = 0.502). Table 3 reports the regression coefficients for the significant model in the “card” group. We can see that the most important predictors were abs, abs_diff, and DET (though the last one did not reach significance). This means that participants reported greater satisfaction with the interactions (a) that contained less overall movement (abs), (b) in which clear roles were established (one person moving more than the other; abs_diff) and (c) in which synchronization episodes were not too long (DET). A similar analysis was performed for the relation between movement characteristics and performance (participants’ satisfaction was included as an additional variable). The model was also significant in the group with the card (*F* = 3.23, df = 8;10, *p* = 0.043) and not in the group without the card (*F* = 0.71, df = 8;12, *p* = 0.681). We report the coefficients of the significant model in the group with the card in Table 4. Significant variables included the recurrence rate and the longest vertical line. This translates to lower synchronization between participants (RR) and higher overall stability (V_max).

**TABLE 3:** Coefficients of the linear model predicting participants’ satisfaction in the group with the card.

	**Standardized coefficient**	**SE**	***t***	***p***
(Intercept)	–0.32	0.12	–2.65	0.023^∗^
RR	–0.22	0.29	–0.75	0.467
DET	–0.90	0.47	–1.94	0.078
DET/RR	–0.48	0.28	–1.72	0.114
LAM	0.42	0.36	1.19	0.260
V_max	0.47	0.31	1.51	0.159
abs	–1.08	0.22	–4.90	< 0.001^***^
abs_diff	1.16	0.23	5.00	< 0.001^***^

*The results of the Wald test for the significance of individual variables are reported (degrees of freedom of *t* statistic df = 11). Significance levels are marked with asterisks: ^∗^*p* < 0.05, ^∗∗∗^*p* < 0.001.*

**TABLE 4:** Coefficients of the linear model predicting task performance in the group with the card.

	**Standardized coefficient**	**SE**	***t***	***p***
(Intercept)	0.53	0.36	1.49	0.167
RR	–2.00	0.70	–2.89	0.016^∗^
DET	1.34	1.26	1.06	0.313
DET/RR	1.33	0.74	1.67	0.125
LAM	–1.05	0.89	–1.19	0.262
V_max	2.61	0.80	3.27	0.008^∗∗∗^
Satisfaction	0.37	0.70	0.52	0.615
abs	–1.46	0.92	–1.58	0.145
abs_diff	0.75	0.98	0.76	0.462

*The results of the Wald test for the significance of individual variables are reported (degrees of freedom of *t* statistic df = 10). Significance levels are marked with asterisks: ^∗^*p* < 0.05, ^∗∗∗^*p* < 0.001.*

Clearly, movement coordination alone does not allow us to distinguish between pairs with the card and pairs without the card and allows us to predict task-related variables only in dyads with the card. This means that introducing the sommelier card does not visibly alter movement coordination but does change the way that coordination impacts interaction outcomes.

## Discussion

The results of our study revealed systematic differences between the pairs using the sommelier card and those who conversed freely without any aid. This cultural artifact can be said to impinge at the individual and systemic levels, influencing (I) the organization of individual behavior, both in the frequency distributions of particular events and in their sequential organization, (II) the coordination of partners on the level of actions performed, and (III) the relation between movement coordination within dyads and the outcome variables of the experiment (the overall performance and satisfaction with the interaction).

(I) At the individual level, participants using the sommelier card employed the olfactory modality more extensively: wine smelling occurred more frequently than in the “no card” condition, whereas wine drinking occurred less frequently. Contrary to what was expected, the overall predictability of behaviors in a sequence did not increase when people were using the card; thus, we cannot interpret the card as a simple constraint that reduces the degrees of freedom of the system. The card does, however, make some transitions between behaviors more frequent than others (controlling for the increased frequency of “smell”). It makes participants less likely to start an interaction with a sample of wine by drinking it than participants without the card and more likely to start an interaction by smelling the wine. The card seems to also make it more probable that the wine will be changed after only being smelled, indicating that participants may prefer to first compare the wines in a single modality (smell) before passing to another modality (taste).

These results can be interpreted as the influence of the professional tool, which codifies not only vocabulary but also the procedure for tasting, describing, and recognizing wines. The card suggests to participants a specific order of behaviors related to wine tasting. However, we found that sequences of behaviors had lower mutual information in the “card” than in the “no card” condition, thus appearing to be less structured. This result was surprising and has verified the way to think about constraints in this situation. We thought (initial hypothesis) that spontaneous tasting would be less structured than tasting with the card. But the card made more frequent the very behavior (smelling) that was underrepresented in the group without the card. Thus, on the level of general entropy – due to encouraging new possibility – we have more equal distribution over the states. This shows that a simple inference from general entropy to behavioral structure complexity might be misleading. In this context we can conclude that the card does not constrain but rather creates new possibilities for participants. These possibilities are akin to the wine tasting strategies employed by professional sommeliers; thus, the sommelier card successfully transferred the embodied behavioral knowledge of wine drinking culture to the naive participants.

(II) At the collective level, we observed a certain degree of coordination of behaviors in both conditions. When drinking wine, pairs with the card displayed a greater amount of role asymmetry than pairs without the card. One of the aspects of asymmetry might be a tendency to establish a leader–follower relation. Participants following the guidelines given by the card drank their wines in a measured and deliberate fashion, focusing also on coordinating their behavior with that of their partner. Thus, we may conclude that the card changes not only the individual behaviors of the participants but also the relation between them.

Such structuring of the interaction, establishing roles and distributing the workload, can be considered a form of adaptation to the demands of joint-action tasks (Marsh et al., 2009; Knoblich et al., 2011; Dale et al., 2013). Indeed, in the original study by Zubek et al. (2016), pairs with the card tended to perform with decreased variance error. The role of the card as a modifier of relations is also consistent with the results of the original study, which found that the presence of the card modified the wine recognition performance of pairs only and not of participants tasting wines individually. Curiously, the observed differences in coordination concern only drinking and not smelling. This might be connected to culturally embodied practices concerning wine tasting that are familiar to the participants. There is a widespread custom of synchronous drinking – making a toast – on various occasions, while no equivalent practice exists for smelling. Additionally, among some pairs without the card, smelling behaviors were so rare at the individual level that it was impossible to measure their coordination in a meaningful way.

(III) Finally, no significant differences between conditions were found concerning low-level movement coordination, but it occurred, that the card acted as a moderator altering relations between movement coordination and two other variables: task performance and reported satisfaction with the collaboration. It can therefore be said that even if the amount of movement is not specific to the system as a whole, the characteristics of the movement gain some functional meaning in the presence of the artifact. The content of participants’ interactions in the two groups was qualitatively different, as the proportions of smelling and drinking changed, but those changes were not apparent in the low-level movement analysis. Among pairs with the card, satisfaction with the collaboration was negatively associated with the overall amount of movement and positively associated with the asymmetry in the activity of the participants, while task performance was negatively associated with the overall amount of coordination and positively associated with the presence of long episodes of repeated movement. These results strengthen our claim regarding the importance of structure and established roles. While raw movement synchrony is reported to be positively correlated with affiliation (Hove and Risen, 2009), Abney et al. (2015) demonstrated that it is weaker coupling (less synchrony) that predicts performance in a structured task involving the manipulation of physical objects and suggested that role asymmetry may also be beneficial in such tasks. This may explain why we observed significant effects of movement coordination only among pairs with the card, as this condition imposed more structure and required the sharing of physical items (cards) between participants.

Summarizing and attempting to generalize, we could say that in terms of degrees of freedom, the sommelier card increased the number of degrees of freedom at the individual level (introducing new behaviors) and constrained degrees of freedom at the collective level (structuring coordination). Recall that in the original study (Zubek et al., 2016), pairs with the card were characterized by smaller variance in their answers and more concise vocabulary in their linguistic interactions. These are all facets of the same tendency of the reduction of degrees of freedom at the collective level. The observation that, at the individual level, degrees of freedom seem to increase explains why in the original study the sommelier card did not reduce the variance of answers in the individual conditions.

We stress that this complex picture in which the presence of a sommelier card opens some possibilities while restricting others should be seen as natural for social and cultural phenomena studied in ecological settings. Complex systems by definition cannot be reduced to simple unidirectional, linear relations. Our findings – clarifying the results of the previous study – were made possible by the choice to look for structured behavior on multiple different levels using different operationalizations.

## Conclusion

Radically embodied perspectives on the development of skills and expertise underscore the role of the acting body in a structured environment. Both the formation of the body through repetitive practice and the progressive modification of the environment are crucial for ratcheting the effects of learning, which is understood as embodied “enskillment” (Ingold, 2000). While the former acts on the developmental and learning time scales, the latter concerns the cultural accrual of expertise.

To understand how the modification of the niche aids in the preservation and propagation of skills, an important task would be to study how this niche, including artifacts, impinges on and controls the embodied practice. This was the main aim of this research: to determine how a culturally created tool for wine description and recognition changes the actual individual practice of such tasks and the coordination of this practice in collective settings. We believe that designing strategies to study such influences in a more systemic way, taking into account their situated and embodied aspects, is an urgent task given the plethora of increasingly technologically advanced artifacts that transform our daily practice and interactions, often in an irreversible way.

In our research, we sought to integrate the sociological, anthropological, and psychological perspectives. The former allowed us to treat artifacts in more active and agentive ways than psychology traditionally permits. Artifacts have the power to change the practice of individuals and create novel relations among them, because they are elements of distributed cognitive systems carrying the intentionality of their makers. In building this integratory framework, we drew on ecological psychology as conceptually helpful to account for the shaping of artifacts as part of the cognitive niche and, in turn, for their role in promoting certain behaviors as individual and interactive affordances within social events. This interdisciplinary approach also facilitates the integration of qualitative and quantitative methods from anthropology, psychology, and the dynamical systems approach in an attempt to first identify the crucial factors and behaviors and then to operationalize the expected effects in a measurable way.

The results of this research testify to the utility of both qualitative analysis and dynamical systems methods, in which the analysis of degrees of freedom on various levels and the examination of systemic stability, variability, and complexity allowed a comprehensive picture of the artifacts’ role to be formed. The sommelier card opened some possibilities at the individual level, bringing into focus modalities and behavioral organizations more in line with professional practice, but also seemed to constrain the collaboration by creating new relations between participants. However, we are aware that both this research tackling the specific problem of wine tasting as embodied practice and the general problem of how to study embodied and situated learning in all its complexity require much further work.

The next concrete step would be to compare the novices in our study to professionals to see if the changes are indeed toward the more skilled practice. The coordinative role of the card may also differ in the professional pairs. Qualitative research on the phases of the task could provide more insight into the ability of the artifact to create specific relations. An important lesson from this study that we hope will continue to inform our research is the conviction that focusing on a single level would not allow us to appreciate the complexity of the studied phenomena. Individuals co-create collective systems in an embodied practice and this can be studied at several levels of organization and using multiple types of observables, from coded behaviors and their frequency and timing, to task performance and assessments of satisfaction with the interaction, to automatic movement analyses. In such an embodied and systemic view, cultural artifacts are considered to have some kind of agency, changing the behaviors of other actors and the relations among them, co-creating social reality.

On a more methodological, final note, the next steps addressing the development of methods to study skill acquisition within the embodied situated and distributed perspectives will involve conceptual work on how to integrate the methods and domains within an explanatory pluralist approach (Abney et al., 2014) in which different scientific disciplines lend their insights and methods to understand the studied phenomena on different scales and at different levels of organization.

## Data Availability Statement

All datasets generated for this study are included in the article/Supplementary Material.

## Ethics Statement

The studies involving human participants were reviewed and approved by the Polish Academy of Sciences Ethics Committee, Institute of Psychology. The patients/participants provided their written informed consent to participate in this study. Written informed consent was obtained from the individual(s) for the publication of any potentially identifiable images or data included in this article.

## Author Contributions

JR-L, JZ, and MD designed the study. JK and NK coded the data. JZ, MD, and KZ performed the behavioral analyses. MK performed the movement analyses. JR-L, JK, NK, KZ, MD, MK, and JZ wrote the manuscript.

## Conflict of Interest

The authors declare that the research was conducted in the absence of any commercial or financial relationships that could be construed as a potential conflict of interest.

## Footnotes

1 An additional asset of focusing on such frameworks is that they diminish the gap between cognitive approaches and the social sciences: “Because cognitive structures need not exist only in the mind [and perhaps never do so, if the radical version of d-cog (distributed cognition) is correct], but instead can exist in the complex interactions of social groups and technological artifacts, one can study social groups cognitively, or cognitive systems sociologically. There need be no unbridgeable divide between social and cognitive explanations” (Brown, 2011, p. 21).

2 One pair with a card was excluded, as in Zubek et al. (2016), because they demonstrated a high level of knowledge about wines.

3 In the initial coding, we decided to add the category of holding the cup with water or wine to investigate if this variable differentiated the examined groups (with and without a card) in some way and if the total contact time with the cup was a variable affecting the performance of the task in any way. We also created the category of marking the cup, which had three subcategories: marking the cup for the first time, changing the previous mark and rejecting the previous mark. However, in the final coding, the category “changing cup” better reflected action organization than “holding cup.” “Marking” was a rare event and did not enter this line of analyses.

4 https://github.com/zubekj/pixel_tracking

5 Note that it is the experimental condition that affects movement coordination and not the other way around, so this is a case of anticausal modeling. We use logistic regression as a multivariate analog of simple correlation, where the direction of the relation is not specified.

## Supplementary Material

DATA SHEET S1 Sommelier Card (original and translation).

DATA SHEET S2 Participants’ Questionnaire (original and translation).

DATA SHEET S3 Raw data files (descriptions in README.txt files).
