# Supplementary material for: Cultural Artifacts Transform Embodied Practice: How a Sommelier Card Shapes the Behavior of Dyads Engaged in Wine Tasting
Source: Front Psychol. 2019 Dec 6;10:2671. doi: 10.3389/fpsyg.2019.02671 (PMC6915083; doi:10.3389/fpsyg.2019.02671)
Supplement: DATA SHEET S1 — Sommelier Card (original and translation). [file Data_Sheet_1.pdf]

OCEŃ WINO ZAKREŚLAJĄC PASUJĄCE ODPOWIEDZI:

Zapach wina

Intensywność zapachu:
słaby – mało intensywny – średnio intensywny – intensywny – bardzo intensywny

Jakość zapachu:
zwyczajny – subtelny – wyrafinowany

Złożoność zapachu:
prosty (wyczuwalne 1-2 zapachy) – złożony (wyczuwalne więcej niż 2 zapachy)

Wyczuwane zapachy (zakreślić i/lub podać własne):
kwiatowe, owocowe, roślinne (np. trawa, mech), korzenne (przyprawy), mineralne, chemiczne, piwniczne
Inne ____

Komentarze:____

Smak wina

Intensywność smaku:
słaby – mało intensywny – średnio intensywny – intensywny – bardzo intensywny

Długość smaku (jak długo smak pozostaje w ustach):
krótko – średnio – długo

Jakość smaku:
zwyczajny – subtelny – wyrafinowany

Obecność cukru:
wytrawne – półwytrawne – półsłodkie – słodkie

Kwasowość:
brak ("płaskie") – lekko kwaskowe (dość świeże) – kwaskowe (świeże) – kwaśne

Alkohol:
słabe – średnie – mocne

Taniny (Garbniki) (szorstkość, uczucie ściągające w ustach):
brak – mało garbnikowe – średnio garbnikowe – mocno garbnikowe

Mineralność:
mdłe – smaczne – słone

Struktura:
słaba – średnia – bogata – ciężka

Komentarze:____

Ogólne własności wina:

Harmonia zapach/smak:
mało harmonijne – harmonijne

Ewolucja:
młode – dojrzałe – stare (przejrzałe)

Komentarze:____

RATE THE WINE BY CIRCLING THE MATCHING ANSWERS:

Smell of wine

Fragrance intensity:

weak - mildly intensive - moderately intensive - intensive - very intensive

Fragrance quality:

ordinary - subtle - sophisticated

Fragrance complexity:

simple (perceptible 1-2 smells) - complex (perceptible more than 2 smells)

Perceptible smells (circle and / or write down your own):

floral, fruit, vegetable (e.g. grass, moss), root (spices), mineral, chemical, cellar

Other ______________________________

Comments:____

The taste of wine

Taste intensity:

weak - low intensity - medium intensity - intensive - very intensive

Taste length (how long the taste remains in the mouth):

short - medium - long

Taste quality:

ordinary - subtle - sophisticated

Presence of sugar:

dry - semi-dry - semi-sweet - sweet

Acidity:

none ("flat") - slightly sour (fairly fresh) – sour sweet (fresh) - sour

Alcohol:

weak - medium - strong

Tannins (roughness, astringency in the mouth):

none - little tanning - medium tanning - tanning

Minerality:

bland - tasty - salty

Structure:

weak - medium - rich - heavy

Comments:____

General properties of the wine:

Harmony smell / taste:

not harmonious - harmonious

Evolution:

young - mature - old (overripe)

Comments:____
