# Supplementary material for: Cultural Artifacts Transform Embodied Practice: How a Sommelier Card Shapes the Behavior of Dyads Engaged in Wine Tasting
Source: Front Psychol. 2019 Dec 6;10:2671. doi: 10.3389/fpsyg.2019.02671 (PMC6915083; doi:10.3389/fpsyg.2019.02671)
Supplement: DATA SHEET S2 — Participants’ Questionnaire (original and translation). [file Data_Sheet_2.pdf]

Informacje o uczestniku badania

1. Wiek: _______ 2. Płeć: K M

3. Wykształcenie: ____________

4. Czy palisz papierosy: T N

    4a. Jeśli tak, to ile:______________

5. Zadanie rozpoznawania wina oceniasz jako:

    bardzo trudne/trudne/średnio trudne/łatwe/bardzo łatwe

6. Jakie cechy wina były dla Ciebie ważne przy rozpoznawaniu:

____

____

7. Jeśli pamiętasz, oceń jak smakowały Ci poszczególne wina:

A: 1----2----3----4----5----6----7

niedobre bardzo dobre

B: 1----2----3----4----5----6----7

niedobre bardzo dobre

C: 1----2----3----4----5----6----7

niedobre bardzo dobre

D: 1----2----3----4----5----6----7

niedobre bardzo dobre

E: 1----2----3----4----5----6----7

niedobre bardzo dobre

F: 1----2----3----4----5----6----7

niedobre bardzo dobre

8. Jakość współpracy przy ocenianiu wina oceniasz:

1----2----3----4----5----6----7

nisko bardzo wysoko

9. W jakim stopniu znasz osobę współuczestniczącą w badaniu:
    1. nie znam
    2. znam w niewielkim stopniu (np. z widzenia)
    3. znam dość dobrze (np. koleżanka/kolega z pracy, znajomy)
    4. znam bardzo dobrze (np. przyjaciel/przyjaciółka, partner)

Information about the participant

1. Age: _______ 2. Sex: F(emale) M(ale)

3. Education: ____________

4. Do you smoke: Y(es) N(o)

    4a. If so, state the amount:______________

5. Do you consider the wine recognition task to be:

    very hard/hard/moderately hard/easy/very easy

6. What qualities of the wines were useful to you during the recognition?

____

7. If you remember, rate how did you like the wines:

A: 1----2----3----4----5----6----7

not tasty very tasty

B: 1----2----3----4----5----6----7

not tasty very tasty

C: 1----2----3----4----5----6----7

not tasty very tasty

D: 1----2----3----4----5----6----7

not tasty very tasty

E: 1----2----3----4----5----6----7

not tasty very tasty

F: 1----2----3----4----5----6----7

not tasty very tasty

8. How would you assess the quality of the cooperation during wine description task:

1----2----3----4----5----6----7

low very high

9. How well do you know your co-participant?

    1. I don't know that person
    2. I know the person very little (eg. have seen before)
    3. I know quite well (eg. acquaintance)
    4. I know very well (eg. friend/partner)
